# The Influence of Adolescents’ Social Networks on Alcohol Consumption: A Descriptive Study of Spanish Adolescents Using Social Network Analysis

**DOI:** 10.3390/ijerph15091795

**Published:** 2018-08-21

**Authors:** Enedina Quiroga, Arrate Pinto-Carral, Isaías García, Antonio J. Molina, Tania Fernández-Villa, Vicente Martín

**Affiliations:** 1 SALBIS Research Group, Faculty of Health Sciences, University of León, Campus of Ponferrada s/n, Ponferrada, 24401 León, Spain; apinc@unileon.es; 2 SECOMUCI Research Groups, Escuela de Ingenierías Industrial e Informática, University of León, Campus de Vegazana s/n, C.P. 24071 León, Spain; isaias.garcia@unileon.es; 3 GIIGAS Research Group, Instituto de Biomedicina (IBIOMED), University of León, Campus de Vegazana s/n, C.P. 24071 León, Spain; ajmolt@unileon.es (A.J.M.); tferv@unileon.es (T.F.-V.); 4 GIIGAS Research Group, Instituto de Biomedicina (IBIOMED), Ciber de Epidemiologia y Salud Pública (CIBERSP), University of León, Campus de Vegazana s/n, C.P. 24071 León, Spain; vmars@unileon.es

**Keywords:** adolescents, alcohol consumption, social-network analysis, structural pattern in the class

## Abstract

In adolescence, friends are important due to their influence on the acquisition of habits such as alcohol consumption. However, there is a lack of studies that describe the structural context of adolescents, which would be useful to implement prevention strategies. Therefore, our research question was how adolescent friendship networks influence alcohol consumption. Our goal was to determine the structural profile of adolescent at-risk alcohol users and their relational context in the classroom. We designed a descriptive cross-sectional study based on social network analysis to analyze structural patterns. We recruited 195 students. Social-network and alcohol-consumption variables were analyzed using the UCINET and STATA programs. Some 86.67% of participants had consumed alcohol at some time in their lives and the prevalence of at-risk alcohol use was higher in females (50.48% vs. 49.52%; OR: 1.84; CI 95%: 0.99–3.43%; *p* = 0.036). The lower the intensity of friendship, the more contacts adolescent at-risk alcohol users had within the network, and the easier it was for them to access their peers. Consequently, we conclude that the structure of a class is a key factor that merits further research.

## 1. Introduction

According to the latest study by the World Health Organization (2018), published as part of the global strategy for reducing the harmful use of alcohol in 2018, levels of alcohol consumption have fallen worldwide in recent years. Nevertheless, alcohol use continues to present a major public health problem, causing many negative biopsychosocial outcomes in the population [1]. It is responsible for 3.3 million deaths worldwide, and not only creates dependency but also increases vulnerability and the risk of numerous diseases [2].

Although alcohol consumption can affect any age group, adolescents are particularly vulnerable, since adolescence is a crucial moment of transition from childhood to maturity, which involves many physical, affective, cognitive, and attitudinal changes [3]. Adolescents’ desire to seek new, intense experiences, expand their social networks, establish their independence, and construct their own identity renders them particularly vulnerable to behaviors associated with alcohol consumption [4].

According to data published by the World Health Organization [5], Spain ranks eighth among European countries for alcohol consumption, and over 75% of the general Spanish population regularly consumes alcohol. Alcohol has become ubiquitous in leisure activities and social relations [6]. As a result, a high percentage of Spanish adolescents report regularly consuming alcohol and having started consumption at a very early age [7]. This presents a high risk to their physical, mental, and social health, and is a clear predictor of possible alcohol dependence and associated risks, as seen in Reference [8]. The Spanish Surveys on Drug Use in Secondary Education (Spanish initials: ESTUDES) indicate that, despite a reduction in consumption rates, alcohol remains the most frequently consumed substance among Spanish adolescents [7]. At European level, the European Monitoring Centre for Drugs and Drug Addiction [9] has also reported that alcohol is the most frequently consumed substance in the adolescent population, followed closely by tobacco and cannabis, noting that polysubstance use is the predominant pattern of consumption in society today.

Polysubstance use increases the risks and exacerbates the negative outcomes of drug use [10]. The two most frequently combined substances are alcohol and tobacco, and the three most frequently combined are alcohol, tobacco, and cannabis, while people who consume four or more substances usually combine alcohol, tobacco, cannabis, cocaine powder and ecstasy or designer drugs [11]. Numerous studies have underlined the risk of alcohol consumption in adolescence, noting that it is associated with established patterns of polysubstance use [12,13].

One of the greatest risk factors associated with alcohol consumption is social influence. This refers to the influence exerted by adolescents’ contacts and social environment. Together with family and peer groups, social interaction, social acceptance, and access to alcohol, they constitute the main factors that contribute to the development of a social environment of alcohol consumption [14]. This is reinforced by a culture in which alcohol is ubiquitous and easily accessible in our social relations and lifestyles [15]. The cultural role and social tolerance and acceptance of alcohol explain why alcohol is the most frequently consumed drug among adolescents [14].

Studies have shown that parents and the population in general are aware that adolescents consume alcohol, but in light of their own past experiences, they view this as a normal part of adolescence [16]. Coupled with an absence of consolidated values in adolescence and the cultural meaning endowed on alcohol as regards to leisure and social integration with the peer group, this lack of social concern currently facilitates the normalization of alcohol consumption [17].

The present study focused on adolescents’ social ties with their peer group within a school environment. As a result of these ties, friendship exerts a strong influence on adolescents’ personal and social development; therefore, identifying how this concept of friendship is constructed would be useful to help formulate preventive strategies against consumption.

The class is the ideal environment for adolescents to develop behaviors and patterns that enable them to interact with their peers [18]. The need to belong and have ties to a group drives adolescents to develop behaviors adapted to the peer group to which they want to belong [19]. Recent qualitative studies [20] have reported an association between adolescents’ social ties in peer groups and alcohol consumption, whereby consumption is directly mediated by peer groups and their relational characteristics. Adolescents form strong ties with their peer groups and, as a result, peer influence contributes to alcohol consumption [20]. Consequently, an analysis of the various influences exerted on adolescents in their socioecological system in the class would help predict many of their harmful habits [21].

However, there is a lack of studies that investigate how these relational structures in the class are constructed, a subject that merits indepth analysis. Unless we know how class networks are constructed and how friendship ties influence substance consumption, we will be unable to tackle a problem that the literature indicates is essentially relational. In this regard, social-network theory concerns social structures and how an individual’s behavior is constructed through their contacts [22]. This conceptual framework formed the fundamental basis of the present study, since it contextualizes the ties that occur in a network and analyzes these groups of ties [22].

The method used to apply this theory and analyze structural data is called social-network analysis (SNA), which enables analysis and interpretation of patterns of behavior [23]. This social and behavioral research approach encompasses a series of methods, models, and applications expressed in terms of relational concepts that can shed light on the kind of ties established between adolescents and their peers and how these can influence their consumption habits [24]. The number of people that form a network, the number of ties that each individual has with the others, the position that each individual occupies within the network, the composition of the network and the ties that are established between members of the network are all extremely important to determine behaviors and the means by which these are propagated [25].

Previous studies have already noted the importance of adolescents’ positions within a network as regards the possible acquisition of behaviors, such as alcohol or tobacco use [21,26]. Although numerous studies have shown that having friends who consume alcohol increases the probability of consumption in the rest of the network [21,27,28,29], few studies have analyzed global class networks (sociocentric networks) in early adolescence.

However, an SNA study of alcohol consumption would shed light on the characteristics of behaviors associated with social structures that may propagate consumption [30,31,32].

Given the foregoing, we have observed a lack of studies that describe the structural context of adolescents, and how adolescent friendship networks influence alcohol consumption. The overall aim of the present study was to determine the structural profile of adolescent at-risk alcohol users and their social and structural context in the class. To this end, we defined the following objectives:

- To determine adolescents’ pattern of consumption in relation to at-risk use of alcohol and other substances.
- To analyze the structural position of adolescent at-risk alcohol users within their peer microsystem in the class.

## 2. Materials and Methods

### 2.1. Participants

Our sample consisted of 195 adolescents aged between 16 and 19 years old who were in their first or second year of postcompulsory secondary education at four public high schools in the region of El Bierzo (León, Spain), in 2017. Parental consent was obtained for all participants, who were recruited through convenience sampling. The total sample consisted of 53.85% females (*n*: 105) and 46.15% males (*n*: 90), who presented a mean age of 17 years old (SD: 0.82). Students in their first year of postcompulsory secondary education accounted for 53.85% of participants, while those in their second year for 46.15%. The socioeconomic data indicated that 39.29% of participants presented a medium-low level, whereas 60.71% presented a high level.

### 2.2. Instruments

We administered an online questionnaire using both validated and ad-hoc instruments to address the research question. Demographic data such as sex, age, and socioeconomic level were collected using the FAS II questionnaire, adding the variables academic year, school, and place of birth. Data on alcohol consumption and the pattern of consumption adolescents acquired in the class were collected using the Alcohol Use Disorders Identification Test (AUDIT) questionnaire for early detection of at-risk use and alcohol-related disorders. This questionnaire is based on a project carried out by the World Health Organization [33], subsequently standardized by Saunders et al. [34] and adapted to Spanish by Rubio [35]. It consists of 10 items that explore the quantity and frequency of consumption, dependent behaviors, and alcohol-related problems. Each item was scored from 0 to 4, with cut-off points set at 8 for men and 6 for women. The AUDIT is a sensitive test (51–97%) to detect the use or abuse risk of alcohol with a high internal consistency (=0.86), as well as sensitivity and specificity values of 90% in both cases [35]. We selected this instrument due to its ease of administration, rapid completion, and precise responses. We also administered a questionnaire similar to the Spanish National Survey on Drug Use in Secondary Education [36]. This questionnaire was used according to the needs of our research to obtain data on polysubstance use and age associated with early consumption.

To collect structural data, we formulated a question about the friendship network, guided by the literature on SNA methodology. To formulate this question, we conducted a review of the literature on the influence of networks on the consumption of alcohol and other substances [37,38,39,40,41,42,43,44,45,46,47]. Thus, the following item was used to ask students about their friendship network: “Using the list below, indicate how much time you spend with your classmates”. Participants ranked the intensity of contact using a scale from 1 (“We never spend time together”) to 5 (“We’re always together”). The list of participants in each class and their responses to this item were used to construct a sociocentric matrix.

### 2.3. Procedure

After receiving approval from the University of León Bioethics Committee (ETICA-ULE-003-2015), we contacted the head teacher at each school to explain the study and seek the informed consent of students and their parents or guardians. The research team agreed with teachers on when to visit the school and administer the instruments in the classroom so as to cause as little disruption as possible to classes. Two researchers visited the schools to administer the questionnaires online. The study involved 72.3% (195/270) of the students from the centers. All the data obtained were stored in an automated electronic file created specifically for this research in compliance with Spanish law (Organic Law 15/1999, of 13 December, on the Protection of Personal Data).

### 2.4. Data Analysis

Data analysis was performed using the STATA 14.0 statistical program, or the UCINET 6.649 program for data of a relational nature [48]. We calculated descriptive statistics to obtain the frequencies and proportions that would enable us to describe sociodemographic characteristics and patterns of drug use in the study sample. We also performed a bivariate analysis to determine the association between structural pattern and alcohol consumption. For all parameters, we calculated a confidence interval of 95% and statistical significance was defined as *p* ≥ 0.05.

For data of a relational nature, we constructed an initial matrix from which we obtained 3 adjacency matrices based on 3 different dichotomization criteria (Table 1) using the scores assigned to time frequencies (1: We never spend time together, 5: We’re always together). Thus, we obtained minimum, intermediate, and maximum contact-intensity matrices [49]. We conducted a descriptive analysis of the matrix data, where the results were the values for the degree of ties surrounding each individual (degree), ties received (indegree), and emitted (outdegree), the degree of proximity (in/out closeness), the degree of intermediation (betweenness), and the level of prestige or influence (eigenvector). All personal information that could identify the students was immediately codified with a fictitious name by the online questionnaire platform in order to ensure anonymity.

## 3. Results

### 3.1. Pattern of Consumption

Of the total sample, 110 students (56.41%) presented risk-free consumption in contrast to 85 students (43.59%) who presented at-risk use. At-risk alcohol use was significantly associated with females, 50.48% of whom presented this pattern of consumption (50.48% vs. 49.52%; OR: 1.84; CI 95%: 0.99–1.43; *p* = 0.036). The mean age of first alcohol consumption in the study sample was 13.4 years old. Some 86.67% of the adolescents reported having consumed alcohol at some time in their lives, compared with 13.33% who had never drunk an alcoholic beverage.

After alcohol, tobacco was the second most frequently consumed substance in our sample, followed by cannabis, sedatives, and stimulants. With regard to the association between the consumption of alcohol and other substances, we found a significant relationship between alcohol and tobacco, and between alcohol and cannabis (OR: 4.62; CI 95% 2.40–8.97; *p* = 0.001) (OR: 4.13; CI 95% 2.03–8.50; *p* = 0.003). The mean age of first use of tobacco and cannabis was 14 and 14.6 years old, respectively.

### 3.2. Structural Position in the Class and Alcohol Consumption

**Figure 1:**
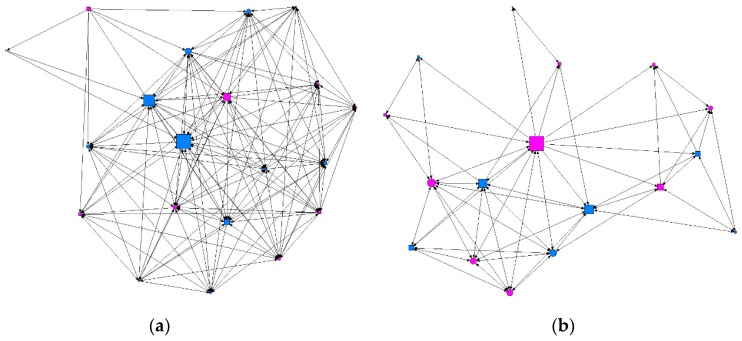
Graph of minimum contact intensity in a class. Males are shown in blue and females in pink; circles represent individuals whose consumption did not present a risk and squares represent individuals showing at-risk use. Graphs produced using UCINET software [48]: (**a**) where the size of the nodes indicates the degree of intermediation (betweenness); (**b**) where the size of the nodes indicates the degree of ties (degree).

**Figure 2:**
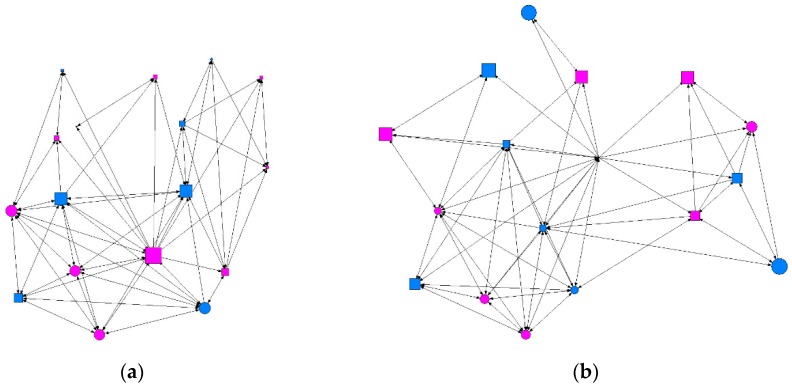
Graph of minimum contact intensity in a class. Males are shown in blue and females in pink; circles represent individuals whose consumption did not present a risk and squares represent individuals showing at-risk use. Graphs produced using UCINET software [48]: (**a**) where the size of the nodes indicates the degree of prestige or influence (eigenvector); (**b**) where the size of the nodes indicates the degree of proximity (closeness).

**Figure 3:**
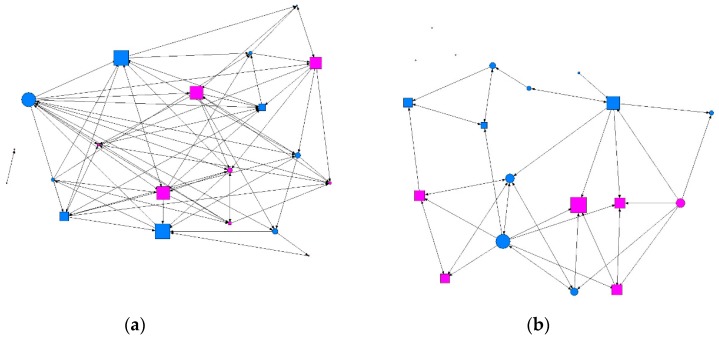
Graph of intermediate contact intensity in a class. Males are shown in blue and females in pink; circles represent individuals whose consumption did not present a risk and squares represent individuals showing at-risk use. Graphs produced using UCINET software [48]: (**a**) where the size of the nodes indicates the degree of intermediation (betweenness); (**b**) where the size of the nodes indicates the degree of ties (degree).

We conducted a descriptive analysis of the matrix data, where the results were the values for the degree of ties surrounding each individual (degree), ties received (indegree) and emitted (outdegree), the degree of proximity (in/out closeness), the degree of intermediation (betweenness), and the level of prestige or influence (eigenvector). Table 2 gives a summary of the descriptive data obtained according to the intensity of contact between individuals: minimum, intermediate, or maximum. We found that at the minimum level of contact intensity, students presenting at-risk alcohol use occupied more central positions in the network, as shown in Figure 1 and Figure 2. At the intermediate level of contact intensity, we observed significant differences in emitted ties, or outdegree, and betweenness centrality, whereby students presenting at-risk alcohol use and an intermediate level of contact intensity in their class friendship network obtained a structural pattern of intermediary, according to the perspective of SNA (Figure 3). No associations with at-risk alcohol use were detected at the maximum contact intensity level.

## 4. Discussion

The overall aim of the present study was to determine the structural profile of adolescent at-risk alcohol users and their social and structural context in the class. 

In order to establish an explanatory framework for our research, one of our goals was to determine the patterns of consumption in the adolescent population through the prevalence of at-risk use of alcohol and other substances. Results show that half of the sample is alcohol-risk consumers, with a significantly higher risk associated with women, a finding in line with the literature [7,9], indicating that alcohol is the most widely consumed substance among adolescents. Our observation of the relationship between alcohol consumption in adolescents and sex is consistent with the findings reported in Spanish Observatory of Drugs and Drug Addiction [7], whereby alcohol consumption is higher in males than in females, but at-risk use is higher in females. In agreement with the data published by ESTUDES [7], our results indicated that alcohol was consumed by a high number of female adolescents and that the percentage of females who got drunk or indulged in binge drinking was higher than that of males.

Our analysis also showed that alcohol, tobacco, and cannabis were the most frequently consumed substances in our adolescent sample. The same results have been obtained in studies by Mccabe et al. [50] and McKelvey et al. [51,52], who found that the most frequently consumed drugs in adolescence were alcohol and tobacco, and that, as adolescents grew older, gaining access to greater financial resources, they began to consume illegal substances such as cannabis. Mccabe et al. [50] and McKelvey et al. [51,52] also observed that single-substance use is practically nonexistent in present-day society, and that the prevailing pattern is instead the use of various substances in conjunction. Hence, there is an association between alcohol use and the consumption of tobacco and cannabis, indicating the existence of a strong relationship between the consumption of so-called conventional drugs and cannabis [53]. This has been corroborated by numerous studies and even by explanatory theoretical models of drug consumption, which show that the use of legal drugs (alcohol and tobacco) is an important prior condition for subsequent consumption of cannabis [54].

The main goal of the present study was to determine adolescent consumers’ structural pattern in the class. The social environment plays a major role in adolescent alcohol consumption [55,56,57,58], since the act of drinking is socially accepted and even useful for social integration [59,60,61,62]. Given that this context always involves ties between individuals, we studied adolescents’ social structure in the class in relation to at-risk alcohol use. Thus, we analyzed the pattern of ties in adolescents presenting at-risk use according to the intensity of contact they maintained with their peers. Our results showed that, at the minimum contact intensity level, at-risk alcohol use was associated with having more contacts and enjoying greater ease of access to peers. In other words, adolescents presenting at-risk alcohol use and minimum contact intensity with their peers tended to connect more easily with the rest of the network than those who did not present at-risk use. Individuals presenting at-risk alcohol use and associated with a higher number of contacts appeared as sociable (degree), close (closeness), and prestigious (eigenvector) to the rest of the network and served as a bridge or intermediary (betweenness) for other contacts.

At the intermediate level of friendship intensity, at-risk alcohol use was associated with having fewer contacts. From this, it follows that at-risk alcohol consumers with intermediate contact intensity sought popularity and sociability (indegree) and attempted to serve as intermediaries (betweenness) with the rest of the network. At the maximum contact intensity level, at-risk alcohol consumers were associated with an absence of contacts and greater difficulty in accessing the network. 

These results are consistent with previous studies reporting that, when friendship criteria are more flexible (comparable to our minimum contact intensity), consumers are considered more fun and have more prestige, and may even occupy leadership roles within the network [63]. Therefore, drinking alcohol confers significant social recognition, but only in cases where the peer group is not bound by strong ties. This finding seems very important and contradicts the notion that drinking occurs among close friends. One explanation may be the negative that occurs in friendship networks with strong ties (comparable to our maximum contact intensity), since individuals with these ties can show an attitude of rejection to alcohol consumption. It is not easy to maintain a real friendship with a person who consumes alcohol, and even less so when at-risk use is involved, since this type of friendship establishes certain limits on actions and thus does not encourage behavior directed towards consumption in adolescents [64].

Numerous studies have linked the existence of alcohol consumption among adolescents with attempts to gain more popularity. Adolescent consumers are socially rewarded by their peers, and blind to the consequences arising from the use of alcohol [20,43,65,66]. In this regard, several studies have analyzed adolescent friendship ties in relation to alcohol consumption from a sociocentric perspective, finding that drinkers are positively associated with centrality measures such as the eigenvector [20,43].

## 5. Conclusions

Our results contribute to the literature on the relational pattern of adolescents in the class and the association between this and alcohol consumption. Adolescents are characterized by being highly influenced by their social environment; consequently, they influence their contacts and are, in turn, influenced by them. The structural pattern associated with alcohol consumption indicates a pattern of higher sociability at lower levels of friendship. Hence, our results are consistent with those obtained in other studies, which have observed this influence or propagation, noting that adolescents presenting at-risk alcohol use can pass on their habit to other adolescents. Adolescents presenting at-risk alcohol use adapt their behavior to contact intensity. Thus, their relational pattern is adapted to their peers according to the intensity of the contact they maintain with these.

Schools offer adolescents a stage of personal growth, expectations, projects, and new challenges. Adolescents begin to establish new contacts with their peers and with members of the opposite sex, which involves adapting to their new social environment. Since the habits adolescents acquire during this stage may have an impact on them in adulthood, for example, creating serious health problems, it is necessary to quantify the structure of social influences in order to tackle this problem effectively. SNA is a useful tool for determining the structural patterns of adolescents. Knowledge of their networks facilitates the design of multifactor strategies targeting the environment and the development of preventive strategies to reduce the negative impact of alcohol on young consumers, not only in terms of their individual health but also in terms of the social and family environment, thus helping to reduce the impact of these problems on the health and welfare system.

Consequently, we would like to highlight the importance of SNA as a method to obtain information on the structural patterns of ties between adolescents. Knowledge about adolescent behavior in peer networks and the influence of the environment could generate solutions to establish collective strategies and combat this important public health problem.

Knowledge in network key would facilitate the planning of preventive strategies that reduce the negative impact of alcohol on young consumers, not only at the level of their individual health, but also in the social environment. Being a determinant for future actions that guarantee better quality of life conditions and improvements in the professionals involved in adolescents’ health.

### Limitations

This study presented the following limitations: (i) The cross-sectional nature of our study prevented us from establishing causality in the associations found; hence, it would be interesting to replicate the study and include longitudinal follow-up to obtain more robust results. (ii) The validity of self-report data cannot be verified, and these should therefore be interpreted with caution since there is a possibility of response bias due to the effect of social desirability, despite the guarantees of anonymity provided.

## Figures and Tables

**Table 1:** Dichotomization by contact intensity.

Contact Intensity	Values Indicating No Contact	Values Indicating Contact
Minimum	1	2, 3, 4, and 5
Intermediate	1 and 2	3, 4, and 5
Maximum	1, 2 and 3	4 and 5

1: we never spend time together; 2: we sometimes spend time together; 3: we spend quite a lot of time together; 4: we’re almost always together; and 5: we’re always together.

**Table 2:** Descriptive statistics for relational variables according to contact intensity between individuals.

Centrality Under Contact Intensity		Mean	SD	CI 95%	P25	P50	P75
**Minimum contact intensity**	**Outdegree**	42.33	1.88	38.62–46.03	29.73	47.06	100
	**Indegree**	42.33	1.42	39.52–45.14	32.45	47.07	90
	**Degree**	57.04	1.060	53.87–60.20	45.02	63.22	100
	**Incloseness**	45.07	1.44	42.23–47.92	34.26	44.74	90.91
	**Outcloseness**	57.33	1.53	54.30–60.35	53.33	63	100
	**Betweenness**	3.27	0.32	2.65–3.90	1.21	3.28	23.3
	**Eigenvector**	30.25	0.75	28.76–31.74	25.68	34.38	55.93
**Intermediate contact intensity**	**Outdegree**	20.26	1.23	17.84–22.68	10.53	20.53	80.14
	**Indegree**	20.26	0.98	18.33–22.19	12.50	25	55.30
	**Degree**	28.39	1.22	25.98–30.81	20	33.33	80.14
	**Incloseness**	20.91	1.022	18.89–22.93	11.25	23.75	60.61
	**Outcloseness**	26.76	1.44	23.92–29.61	15	30.32	83.43
	**Betweenness**	4.46	0.47	3.54–5.39	0.60	4.48	33.26
	**Eigenvector**	26.82	1.25	24.34–29.30	16.18	37.25	69.97
**Maximum contact intensity**	**Outdegree**	11.04	0.83	9.41–12.68	5	12.88	47.26
	**Indegree**	11.05	0.68	9.71–12.38	5.56	15	40
	**Degree**	11.04	0.68	9.71–12.38	5.56	15	40
	**Incloseness**	9.69	0.44	8.82–10.57	6.02	9.17	25.34
	**Outcloseness**	11.04	0.65	9.75–12.33	5.88	9.09	37.21
	**Betweenness**	3.02	0.44	2.14–3.89	0	1.96	28.52
	**Eigenvector**	23.17	1.59	20.04–26.30	5.82	30.90	76.10

Degree: the ties surrounding each individual; indegree: ties received; outdegree: ties emitted; in/outcloseness: degree of proximity; betweenness: degree of intermediation; eigenvector: degree of prestige/influence.
